# Supplementary material for: Asymmetric spin echo multi-echo echo planar imaging (ASEME-EPI) sequence for pre-clinical high-field fMRI
Source: bioRxiv. 2024 Oct 13:2024.10.12.617985. Preprint. [Version 1] doi: 10.1101/2024.10.12.617985 (PMC11482890; doi:10.1101/2024.10.12.617985)
Supplement: Supplement 1 [file NIHPP2024.10.12.617985v1-supplement-1.pdf]

bioRxiv preprint doi: https://doi.org/10.1101/2024.10.12.617985; this version posted October 13, 2024. The copyright holder for this preprint (which was not certified by peer review) is the author/funder, who has granted bioRxiv a license to display the preprint in perpetuity. It is made available under aCC-BY 4.0 International license.

Supporting information

S1 Appendix. Signal Intensity Derivation. Signal intensity comparison between ASEME-EPI and GRE-EPI.

S1 Table. Empirically determined α's ($\alpha_{empirical}$) for various family-wise error (FWE) rates. The $\alpha_{empirical}$'s were calculated in a single subject. Each $\alpha_{empirical}$ is rounded to three decimal places for improved readability.

S2 Table. Empirically determined α's ($\alpha_{empirical}$) for a set family-wise error (FWE) rate equal to 0.01. The number of active voxels exceeding $\alpha_{empirical}$ in the brain is reported in parentheses. Each $\alpha_{empirical}$ is rounded to three decimal places for improved readability.

S1 Figure. Single subject, single slice activation maps. The t-statistic thresholds correspond to the $\alpha_{empirical}$ values cited in S2 Table. Marked voxels are those that exceeded the t-statistic threshold, and are considered active according to the general linear model fit. The various family-wise error (FWE) rates are shown as rows, and are arranged from top-to-bottom as follows: 0.0005, 0.001, 0.005, 0.01, and 0.05. The different acquisitions are shown as columns, and are arranged from left-to-right as follows: GRE, ASEME, SE16, SE40, SEASE, ASE1, and ASE2.
